# Supplementary material for: Low-Energy X-Ray Intraoperative Radiation Therapy (Lex-IORT) for Resected Brain Metastases: A Single-Institution Experience
Source: Cancers (Basel). 2022 Dec 20;15(1):14. doi: 10.3390/cancers15010014 (PMC9817795; doi:10.3390/cancers15010014)
Supplement: Supplementary file 1 [file cancers-15-00014-s001.zip › cancers-2070929-supplementary.pdf]

cancers

Supplementary Materials

Low-Energy X-Ray Intraoperative Radiation Therapy (Lex-IORT) for Resected Brain Metastases: A Single-Institution Experience

Christian D. Diehl, Steffi U. Pigorsch, Jens Gempt, Sandro M. Krieg, Silvia Reitz, Maria Waltenberger, Melanie Barz, Hanno S. Meyer, Arthur Wagner, Jan Wilkens, Benedikt Wiestler, Claus Zimmer, Bernhard Meyer and Stephanie E. Combs

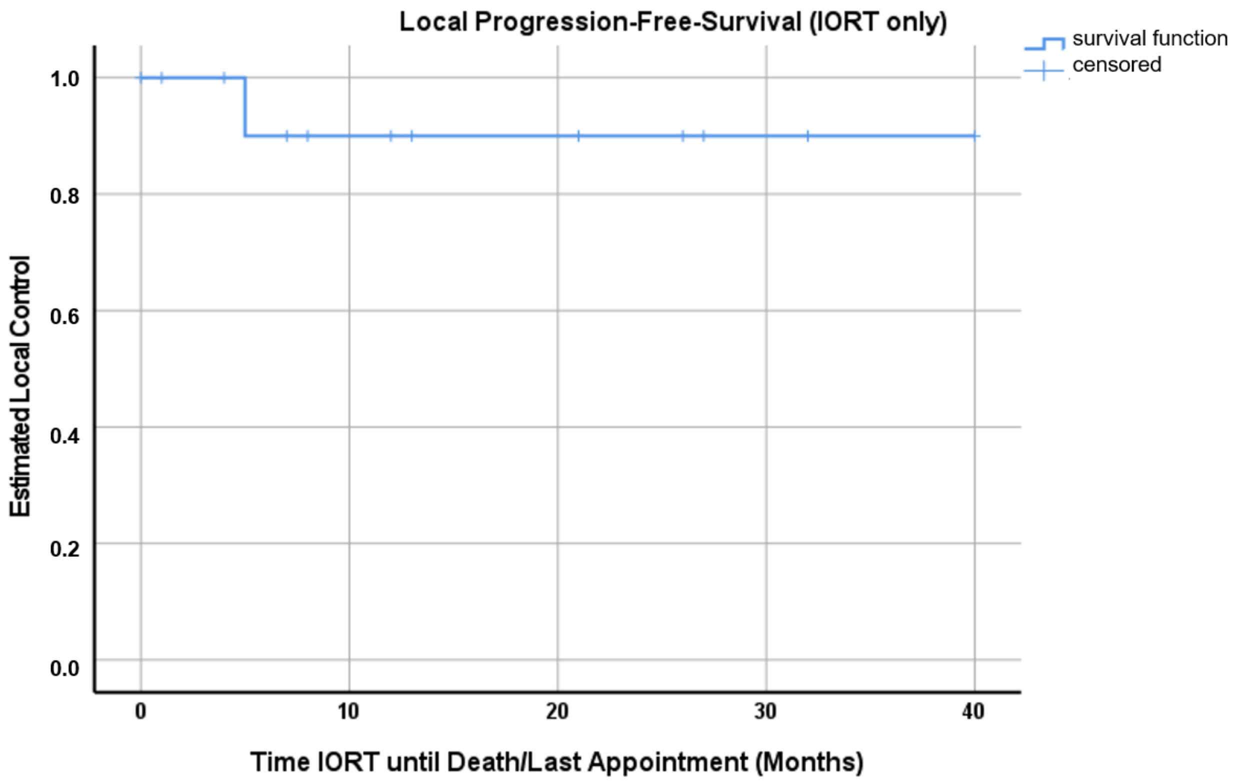

Figure S1. Kaplan-Meyer curve for local progression free survival when excluding patients receiving additional RT post IORT. Estimated LC is 90.0% (95%-CI 71.4 – 100%).
